# Nonword Repetition – A Clinical Marker for Specific Language Impairment in Swedish Associated with Parents’ Language-Related Problems

**DOI:** 10.1371/journal.pone.0089544

**Published:** 2014-02-24

**Authors:** Nelli Kalnak, Myriam Peyrard-Janvid, Hans Forssberg, Birgitta Sahlén

**Affiliations:** 1 Department of Women’s and Children’s Health, Astrid Lindgren Children’s Hospital, Karolinska Institutet, Stockholm, Sweden; 2 Department of Biosciences and Nutrition, Karolinska Institutet, Huddinge, Sweden; 3 Department of Logopedics, Phoniatrics, and Audiology, Clinical Sciences, Lund University, Lund, Sweden; Birkbeck College, United Kingdom

## Abstract

First, we explore the performance of nonword repetition (NWR) in children with specific language impairment (SLI) and typically developing children (TD) in order to investigate the accuracy of NWR as a clinical marker for SLI in Swedish-speaking school-age children. Second, we examine the relationship between NWR, family aggregation, and parental level of education in children with SLI. A sample of 61 children with SLI, and 86 children with TD, aged 8–12 years, were administered an NWR test. Family aggregation, measured as the prevalence of language and/or literacy problems (LLP) in parents of the children with SLI, was based on family history interviews. The sensitivity and specificity of nonword repetition was analyzed in a binary logistic regression, cut-off values were established with ROC curves, and positive and negative likelihood ratios reported. Results from the present study show that NWR distinguishes well between Swedish-speaking school-children with and without SLI. We found 90.2% sensitivity and 97.7% specificity at a cut-off level of −2 standard deviations for binary scoring of nonwords. Differences between the SLI and TD groups showed large effect sizes for the two scoring measures *binary* (d = 2.11) and *percent correct consonants* (PCC) (d = 1.79). The children with SLI were split into two subgroups: those with no parents affected with LLP (n = 12), and those with one or both parents affected (n = 49). The subgroup consisting of affected parents had a significantly lower score on NWR binary (p = .037), and there was a great difference between the subgroups (d = 0.7). When compared to the TD group, the difference from the subgroup with affected parents was almost one standard deviation larger (d = 2.47) than the difference from the TD to the subgroup consisting of non-affected parents (d = 1.57). Our study calls for further exploration of the complex interaction between family aggregation, language input, and phenotypes of SLI.

## Introduction

Children with specific language impairment (SLI) have a deficient language development, without general cognitive delays, physical disabilities, neurological problems, or hearing impairment that can explain their difficulties. SLI is a developmental disorder, characterized by deficits in aspects of language comprehension, production, and function. The prevalence is estimated at 6–7% [1], [2], with a male to female ratio ranging from 2∶1 to 3∶1 ([3], p.38). SLI often entails persistent language problems [4], though the profile of the linguistic difficulties in SLI are dynamic and change with time due to e.g., development, input, and intervention [5], [6].

Since the 1980s, studies of potential clinical markers of SLI have focused mainly on nonword repetition (NWR), [7], verb morphology [8], and lately on sentence recall [9]. Their potential as clinical markers has not yet been studied in Swedish SLI. In the present paper we want to explore sensitivity and specificity of a NWR test in a comprehensive study on school-age children with SLI and controls. It is well known that speech and language impairments are highly heritable [10], [11]. Our second purpose is, therefore, to explore whether there is an association between NWR performance, family aggregation of language-related problems, and parents’ level of education.

Nonwords are made-up words without meaning, constructed according to the phonotactic rules of the target language; for example,“woogalamic” in English [12], and “sallotan” in Swedish [13]. In a NWR test, participants get to hear one nonword at a time, which they are asked to repeat. The repetitions can be scored on the basis of the whole nonword correct or not, or with a more detailed scoring according to, for example, the percentage of correctly produced consonants. The NWR construct is founded in cognitive psychology, and has been claimed to capture phonological working memory capacity constraints in children with SLI [14]. Today, most researchers agree that NWR is a complex task that taps a range of cognitive and linguistic output and input constraints in children with SLI [15]–[18].

Most research of NWR as a clinical marker has been based on English-speaking samples [19]–[21]. NWR has been found to distinguish children with SLI from typically developing children by showing high sensitivity and specificity. Lately, poor NWR has been reported as a clinical marker for SLI in several languages such as Spanish [22], Dutch [23], French [24], Italian [25], and Slovak [26]. Such studies are, however, lacking in Swedish-speaking children.

Most children with SLI are diagnosed at preschool age, though SLI in school-age children is still at risk of not being identified [27], [28]. In Sweden, school focus is indeed on problems related to academic achievement, and especially to reading skills, but due to lack of speech-language pathology expertise within the schools, some aspects of language-related problems are particularly difficult for school staff to discover. The risk of under-identification especially applies to children with receptive language impairments [29], who are also at substantial risk of having social and academic difficulties [30]. Simple and valid screening tools are, therefore, still needed within the Swedish school system.

Several twin studies have reported high heritability in SLI, indicating a strong genetic influence [31]–[33]. Moreover, problems with NWR have been found to be highly heritable and associated with poorer language acquisition [34] as well as genetically linked to chromosome 16 [35], [36]. It is important to remember that heredity for many children with SLI may be associated with a family context with language-related problems in parents, siblings, and grandparents. Family history studies have shown higher prevalence of language-related problems in relatives of children with SLI than in controls [37]–[41]. Growing up with parents affected with language-related problems such as with language or reading impairment for example, probably influences verbal communication in the family, and the linguistic input to the child with SLI. Thus, family aggregation means that language-related problems in parents may be genetically transmitted to the child, but can also influence the child’s home language and literacy environment. The knowledge of how these factors influence language skills in children with SLI is sparse. Previous studies have shown that parents of children with language impairment in general provide simpler, and cognitively and linguistically less demanding input as compared to controls. Also, home reading behavior has been shown to be less extensive in families of children with SLI as compared to controls [42]. There are, however, no studies investigating language input for children with SLI where the parents’ possible language-related problems are considered. Comparisons of language input for children with SLI growing up with affected versus non-affected parents are lacking, as Corrigan [43] points out.

Another factor which may contribute to the home language environment is parental level of education, often used as an approximation of socio-economic status (SES). Parental level of education, or SES, has been suggested to have both a direct and indirect influence on children’s cognitive development [44]. Parents with a higher level of education have been shown to provide not only a linguistically and cognitively more stimulating home environment, but also higher expectations on the child, with a positive influence on language development in typically developing children [45]. Parents to children with SLI have been shown to represent all levels of education [46], and other studies have reported that parents of children referred to speech-language clinics were more highly educated compared to the general population [47], [48].

### Research Questions

Is NWR a clinical marker for Swedish school-age children diagnosed with SLI? Given the high prevalence of language-related problems in parents of children with SLI, is there an association between family aggregation and NWR performance? Finally, is the parents’ level of education associated with family aggregation and NWR performance?

## Materials and Methods

### Ethics Statement

The study is part of a larger research project aiming to describe the linguistic, cognitive, and genetic characteristics of children with SLI, and has been approved by the local ethics committee in Stockholm in Sweden (Reference nos. 2008/543-31/3) and for children with typical development (Reference no. 2012/1938-32).

### Participants

#### Children with SLI

The children with SLI are the same participants as in our previous study [39]. The sample was recruited from all fourteen Stockholm County school language units for children with SLI. These language units are attached to mainstream schools. The general admission requirement for these units is SLI as the primary or only diagnosis; in other words, this is excluding autism and mental retardation, and requires a non-verbal IQ>80. The assessments required for admission are performed by a speech-language pathologist, a psychologist, and a teacher. The following language abilities are assessed by the speech-language pathologist: language comprehension, grammatical production, lexical abilities, phonological output, oral motor skills and social-communication abilities. General cognitive ability is assessed by a psychologist. A teacher will typically observe the child together with peers while in kindergarten. The head of each school was contacted to obtain permission for recruitment of participants, and thereafter we consulted each unit to identify children fitting our study criteria. The schools confirmed that the children recruited for our study still have SLI as their primary diagnosis at the time of participation, and they have been known by the school for at least one year, in most cases longer. One hundred children aged 8–12 years satisfied the study criteria of SLI as the primary diagnosis, with normal hearing and vision according to the parents and the schools, as well as being monolingual Swedish-speaking, and not adopted. The children with SLI were invited by regular mail sent to their parents. Written informed consent was obtained from the parents of 61 children (15 females and 46 males; mean age 9.3, SD 1.2), corresponding to a response rate of 61%. Oral informed consent was obtained from each participant at the time of assessment. The SLI families agreed to participate in a research project comprising cognitive/linguistic assessment of the SLI proband, a family history interview with the parents, and DNA samples from all members of the nuclear family.

The children with SLI had a mean non-verbal IQ of 99.34 (SD 14.4) as measured with Raven’s Colored Matrices [49]. Four of the 61 children with SLI were also diagnosed with ADHD (6.6%), and three were also diagnosed with dyslexia (4.9%). All participants with SLI performed below −1.5 standard deviations on tests of both expressive and receptive language. Speech production was assessed based on a picture-naming task and spontaneous speech during a narrative task; both tasks are part of the comprehensive individual assessment, and will not be further reported here. Speech production was judged as: (1) normal speech status, (2) minor speech deficits; e.g., occurrence of substitutions of/r/, or lisping, (3) occurrence of both context-dependent and context-independent phonological processes i.e., substitutions of consonants or vowels, reductions of syllable structure, reduplication of syllables and assimilations. At the time of inclusion in our study, 40 (66%) children had speech output deficits; half belonging to Category 2 and half to Category 3, and 21 (34%) of the children with SLI had no speech output difficulties.

#### Children with typical development

For the comparison on nonword repetition, a control group consisting of 86 typically developed (TD), monolingual Swedish-speaking children, aged 8–12 years (43 females and 43 males; mean age 9.4, SD 1.3) was used. They were recruited from mainstream schools within a municipality in central Sweden, and had no history of developmental problems according to parents’ and teachers’ reports. Written consent was obtained from the parents of all 86 children with TD. Non-verbal IQ’s were within normal limits (102.4, SD 21.7) as measured with the Block Design Subtest from the WISC-III battery [50]. The children with TD were originally recruited for another project [51].

#### Group comparisons of distribution of age and gender and non-verbal IQ

The children in the SLI and TD groups did not differ significantly regarding the mean age of the participants (p = .538), and the median age was the same in both groups (9.0 years). The SLI and TD groups did not differ significantly regarding the distribution of number of children of each age based on full years (8–12 years), (Pearson Chi^2^, p = .504); this enables group comparisons without need to consider age as a variable. There was a significant difference between groups for the proportion of female to male participants, with the SLI group having 24.6% female participants as compared to 50% in the TD group (Chi^2^ = 8.61, p = .003). The SLI and TD groups did not differ significantly regarding non-verbal IQ (p = .444), based on different tests of non-verbal IQ, as described above. Raven’s Colored Matrices and the Block Design Subtest test were used as proxy for non-verbal IQ, both tests strongly correlate with full scale IQ, and with each other [52].

### Assessment of Nonword Repetition

#### The nonwords

The NWR test is part of the computer-based test battery Sound Information Processing System, or SIPS [51], which was developed based on [17]. The NWR test consists of 24 nonwords, comprised of equal numbers of three and four syllable nonwords. Resemblance to real words was avoided; for example, by not including grammatical morphemes or a stressed syllable that could resemble a real word. Three- and four-syllable nonwords were balanced in terms of stress pattern and number of nonwords having a consonant cluster; half of the nonwords having a cluster are constructed according to Swedish phonotactic rules, and half violate these rules (Table 1).

**Table 1:** Descriptive information about the 24 nonwords.

Nonword length	Consonants	Iamb/Trochee^a^	Cluster^b^	Non-Swe clusters^c^
**Three syllables (12)**	53	6/6	8	50%
**Four syllables (12)**	67	6/6	8	50%

a Stress pattern; number with iamb and trochee.

b Number of nonwords with a consonant cluster.

c Percentage clusters not following Swedish phonotactic rules.

#### Assessment procedure

In both groups, the assessments of NWR were part of a more comprehensive battery of cognitive and linguistic tests. Each participant was assessed individually in a quiet room at their schools. The nonwords were presented digitally from a portable laptop computer, by a female speaker voice, with a central Swedish dialect. The children were told they would hear some made-up words without meaning, one at a time. The children’s responses, that is, their repetitions of the nonwords, were transcribed online and audio recorded for later analysis of reliability. The duration of the NWR testing was 10–12 minutes per child.

#### Scoring

The responses were scored both binary as either correct or incorrect for each of the 24 nonwords (NWR Binary), and as “percent consonants correctly” (PCC) reproduced of a maximum of 120 consonants in the 24 nonwords (NWR PCC), namely the percentage of consonants reproduced correctly and at the correct position in the nonword. As developmental speech errors are normally not present in Swedish 8–12 year old children [53], we only accepted correct repetition of the nonword.

#### Tests of reliability

In order to control for reliability of NWR scoring, a random sample of 18% (11/61) of SLI children’s responses on the NWR was analyzed and scored independently by the first and the last authors of this study, both of whom are speech-language pathologists. The proportion of inter-rater agreement in the SLI group was 100% for NWR Binary, and 96.1% for NWR PCC. In the TD group, all responses were scored and analyzed by a psychologist and a speech-language pathologist. The proportion of inter-rater agreement was 100% for NWR Binary in the TD group. For the NWR PCC in the TD group, there were only a few cases where scoring differed between raters and consensus was reached by discussion. In order to control for reliability of the judgments of speech production (three categories) in the SLI group, a randomly selected sample of 10% of the recordings was analyzed by a research assistant. The inter-rater agreement was 100%.

#### Parents to the children with SLI

The parents of the 61 children with SLI participated in a family history interview [39]. We investigated the prevalence rates of several language-related diagnoses and problems in relatives of the 61 children with SLI and in a control group of 100 typically developing children (not the same controls as in the NWR part of the present study). We asked the parents if they had a history of or current difficulties within several categories of language related diagnoses and problems. We found that the most common problems reported for the parents of the children with SLI were (I) literacy (37.3%) and (II) language (30.5%) problems, both with significantly higher prevalence rates than what we found in the parents of the controls (5% literacy problems, 1.5% language problems). Problems with literacy were classified as difficulties in learning to read and write that were not due to inadequate schooling or bilingualism, and having a diagnosis of dyslexia. Problems with language was classified as difficulty with language acquisition: late talkers (older than 3 years), having received speech/language therapy, and having a diagnosis of developmental language impairment. In the present study, we classified family aggregation into two categories on the basis of whether the child with SLI had parent(s) with language and/or literacy problems, or not. We also gathered information about the parents’ level of education, which was defined in three groups based on their highest level of education achieved: elementary school, upper secondary school, or having pursued higher education/university studies. We have information regarding parental education for all 61 children’s biological parents, except for one father.

Our family history study [39], also incorporated information about the grandparents and siblings. However, in the present study we decided to include information only about the parents. In Sweden, family constellations are usually represented as nuclear families, and do not include grandparents; with other words, grandparents generally do not provide the child with daily linguistic input. In cases where parents are separated, custody is usually shared, with children alternating between parents. Furthermore, we could not control for possible confounding factors regarding the siblings; this includes number of siblings, siblings order, and age of the siblings, as, for instance, siblings can be too young to be present with language and/or literacy problems. Therefore, in the present study, we neither included information about the grandparents nor the siblings.

## Results

We are first reporting performance and group differences on the measures NWR Binary (i.e., percent whole nonword correct) and NWR PCC (i.e., percentage correct consonants) for children with SLI (SLI group), and children with typical development (TD group). Thereafter, we investigate if these measures are showing diagnostic accuracy for children with SLI, both with and without speech output deficits. Further, in the SLI group, we examine the association of nonword repetition to reported prevalence of language and/or literacy problems, and level of education in parents.

### SLI and TD Group’s Performance and Group Differences on Nonword Repetition

One group of 61 children with SLI, and another, consisting of 86 children with TD, completed the NWR test. We found significantly lower results in the SLI group on the NWR Binary and NWR PCC scorings as compared to the TD group. Table 2 shows the results of mean percentages correct for NWR Binary, NWR PCC and NWR length, and the corresponding standard deviations for the SLI and TD groups. Differences between the SLI and TD groups showed large effect sizes for NWR binary and NWR PCC measures (Table 2); the largest was found for NWR Binary: d = 2.11. In the TD group, a tendency of a ceiling effect on NWR PCC was observed. There was no difference between the SLI and TD groups as to NWR length, (i.e., percentage nonwords with correct number of syllables).

**Table 2:** Mean NWR Binary, NWR PCC and NWR Length for the SLI group and TD groups.

NWR measure	SLI (n = 61)		TD (n = 86)		p-value^d^	D^e^
	Mean(SD)	Min-max	Mean(SD)	Min-max		
**NWR Binary** a	26.8% (20.6)	0–75%	64.2% (14.2)	21–96%	.001	2.11
**NWR PCC** b	69.9% (14.4)	32–95%	89.7% (5.9)	71–100%	<.001	1.79
**NWR Length** c	84.5% (14.5)	27–100%	83.6% (10.2)	54–100%	.738	–

a Percentage correct repetition of whole nonwords, 24 items.

b Percentage correct repetition of the 120 consonants in the nonwords.

c Percentage nonwords with correct number of syllables.

d P-values below.05 are reported as significant.

e Cohen’s *d*; effect size for comparison of two means.

### Association of Age, Gender, and Non-verbal IQ with NWR

**Figure 1:**
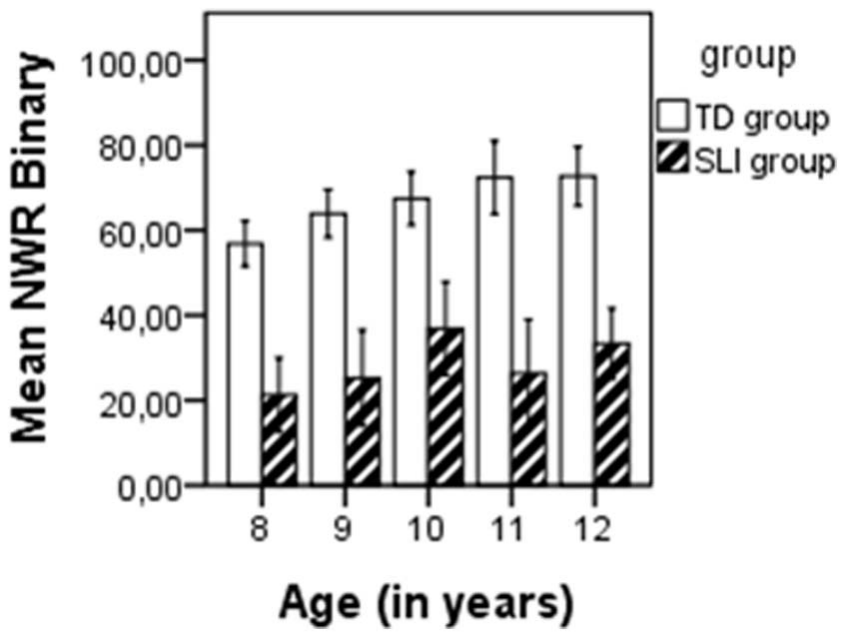
Mean percentage NWR Binary per age, in the SLI and TD groups.

**Figure 2:**
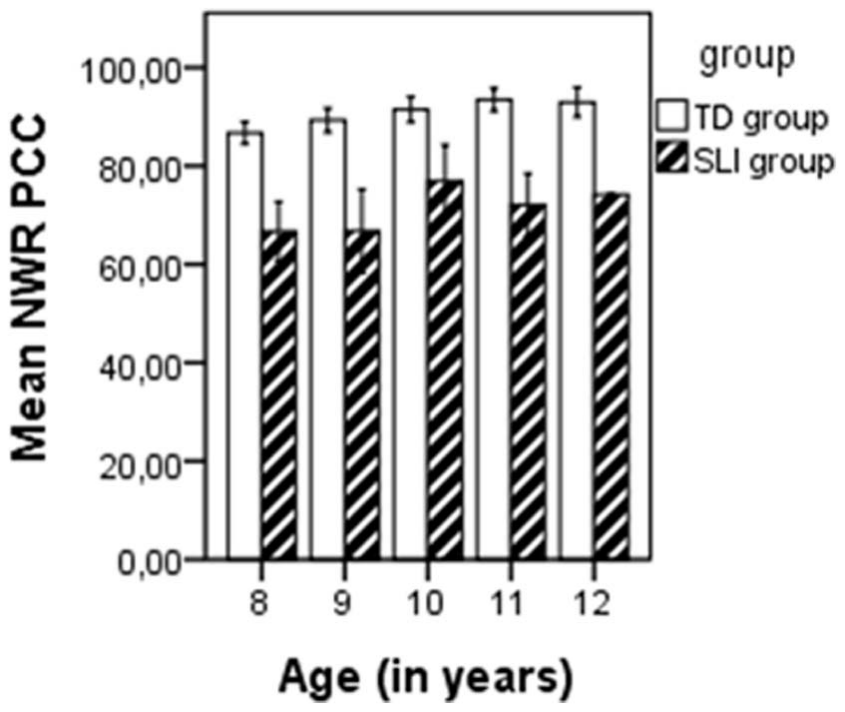
Mean NWR PCC per age, in the SLI and TD groups.

Figure 1 shows the mean percentage NWR Binary, and Figure 2 shows the mean percentage NWR PCC for every age (8–12 years) in the SLI and TD groups, respectively. In the SLI group, there was a non-significant association between age and NWR Binary (p = .067) and NWR PCC (p = .087), as determined by Spearman’s bivariate correlation. However, in the TD group, there was a significant association between age and NWR Binary (r = .393, p = <.001) and NWR PCC (r = .415, p = <.001). There were no gender differences as determined by ANOVA for NWR Binary in the SLI group (F(1.59) = .020, p = .889) and in the TD group (F(1.59) = 0.068, p = .796), and for NWR PCC in the SLI group (F(1.84) = .010, p = .921) and the TD group (F(1.59) = .037, p = .848). There were no significant associations between non-verbal IQ and NWR Binary in the SLI group (p = .146) and in the TD group (p = .127), as well as for NWR PCC in the SLI group (p = .129) and in the TD group (p = .097).

### Clinical Accuracy of Nonword Repetition

Following our aim to investigate the clinical accuracy of the NWR for SLI, we decided to do a bivariate logistic regression analysis. Both NWR Binary and NWR PCC showed non-significant Hosmer & Lemeshow goodness of fit test, as required for each measure included in the analysis. However, we found high associations between NWR Binary and NWR PCC in the entire sample of children with SLI and TD (r = .915, p<.000), in the SLI group (r = .872, p<.000), and in the TD group (r = .811, p<.000). This implies that the two measures are equally adequate to be included in the regression analysis, and that they would need separate analyses. We decided to pursue only NWR Binary in further analysis. NWR Binary is a more reliable and much faster method of scoring than NWR PCC; it is, therefore, a more applicable method of scoring in screening settings. NWR raw scores were converted into z-scores relative to each age (in years) in the TD group. The SLI group’s scores (mean −8.57, SD 5.37) were significantly lower (p = <.001, d = 2.23) as compared to the TD group’s scores (mean 0.03, SD 0.99).

**Figure 3:**
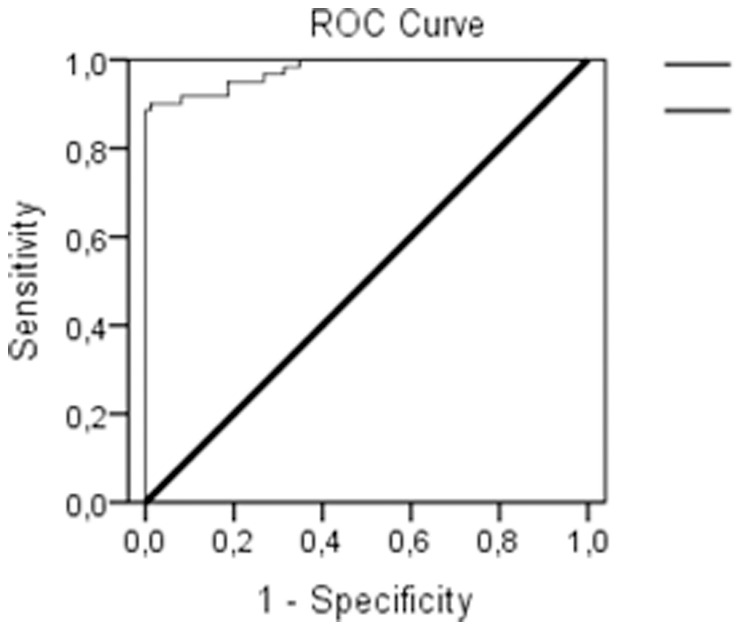
Area under ROC curve.977 (CI.957–997).

The results, based on bivariate logistic regression, proved good ability for the NWR-test to distinguish children with SLI from TD children (Table 3), with 90.2% sensitivity and 97.7% specificity. The best cut-off point based on NWR Binary z-scores was investigated with the response operating characteristics (ROC) curve, and was established at −2.0 standard deviations (Table 3). The area under the ROC was.977 (Figure 3), telling us that the probability of a randomly selected child from the TD group scoring higher than a randomly selected child from the SLI group was 97.7%. Further on, we found a positive likelihood ratio of 38.8 (CI 9.8–152.9); in other words, the odds for a score in the “SLI-affected” range coming from a child in the SLI group and not a child from the TD group, and a negative likelihood ratio of 0.10 (CI 0.05–0.22) for the odds of a score from the “non-SLI” range coming from the SLI group. When inspecting less severe cut-off values at −1.5 and −1.0 standard deviations, we found no change or a small gain in sensitivity, and a loss of up to 10.5% in specificity (Table 3), as well as lower likelihood ratios.

**Table 3:** Diagnostic accuracy for different cut-off values on NWR Binary.

Cut-off	SLI group(sensitivity)	TD group(specificity)	LR+^a^	LR−b
**−2.0 SD**	90.2% (55/61)	97.7% (84/86)	38.8	0.10
**−1.5 SD**	91.8% (56/61)	91.9% (79/86)	11.3	0.09
**−1.0 SD**	91.8% (56/61)	87.2% (75/86)	7.2	0.09

a *Positive likelihood ratio.*

b *Negative likelihood ratio.*

### Clinical Accuracy based on Co-occurrence with Speech Output Deficit

Sensitivity was further analyzed based on speech production of the children with SLI, categorized into three groups (see Methods section), and at a cut-off value of below −2 standard deviations on NWR Binary. We found 75% sensitivity in Category 1 (n = 21) in children with normal speech status; 95% sensitivity in Category 2 (n = 20) in children with minor speech deficits, defined as occurrence of substitutions of /r/, or lisping; and 100% sensitivity in Category 3 (n = 20) in children with occurrence of both context-dependent and context-independent phonological processes i.e., substitutions of consonants or vowels, reductions of syllable structure, reduplication of syllables and assimilations. When adding up Categories 1 and 2, sensitivity was 85.4%; Categories 2 and 3 added together had sensitivity of 97.5%.

### Association between the Performance of the Children with SLI on NWR and Parents’ Prevalence of Language and/or Literacy Problems

The numbers of children with SLI having parent(s) with language and literacy problems (LLP), based on the family history interview, are reported in Table 4. We found that 39 (63.9%) children had parent(s) with literacy problems; clinically diagnosed dyslexia was reported in two of these families, and in the remaining 37 families, the parents had un-diagnosed reading problems. Thirty-eight (62.3%) of the children had parent(s) with language problems. Furthermore, we found that 49 (80%) of the children lived in families with parent(s) having LLP. The co-occurrence of language and literacy problems was high in the parents: 55.3% of the parents with language problems also had literacy problems, and 43.8% of the parents with literacy problems also had language problems.

**Table 4:** Number (%) of children with SLI having parent(s) with language related problems.

Language related problem	YES	NO
**Language**	38 (62.3%)	23 (37.7%)
**Literacy**	39 (63.9%)	22 (36.1%)
**Language and/or literacy**	49 (80.0%)	12 (20.0%)

We went on to investigate the relationship between the SLI group’s performance on NWR Binary z-scores and reported family aggregation. Family aggregation was defined in two subgroups: one with *none* of the parents, and one with *any* of the parents being affected with LLP; these may also be referred to as non-affected or affected parents, respectively.

The SLI subgroup with non-affected parents performed significantly better on NWR (F(1.59) = 4.559, p = .037), as determined by ANOVA, and there was a large difference between the subgroups (d = 0.7), (Table 5). The difference between the TD group and the SLI subgroup with affected parents was almost one standard deviation larger (d = 2.47) than the difference between the TD group and SLI subgroup with non-affected parents (d = 1.57). Three of the children were siblings, and therefore analyses were performed with one sibling at a time to investigate if these three children with the same family history data (both parents affected), yet slightly different results on NWR, had inflated the results. We included all three siblings in the analysis since we found no alteration of results with either all, two, or one sibling at a time being included (p-value varying between.037 and.038). We have investigated the association of literacy problems with language problems, and found that none of these problems are separately related to children’s NWR. It is only when we add up these two family risk components that we find a statistically significant association with NWR performance in children with SLI.

**Table 5:** NWR Binary z-scores for SLI subgroups and children with TD.

SLI subgroup or TD group	N	NWR Binary, mean (SD)
**SLI subgroup with** **affected parent(s)**	49	−9.3 (5.25)
**SLI subgroup without** **affected parents**	12	−5.7 (5.06)
**TD group**	86	0.03 (0.99)

In the subgroup of 49 children with SLI with affected parent(s), 15 of the children had only affected mothers, 18 had only affected fathers, and 16 had two parents affected with LLP. Prevalence of LLP in *both parents* as compared to in only *one of the parents* was not linked to any significant difference on NWR performance. Of the 6 children with SLI performing within the norm average (above −2 standard deviations) on NWR, 50% (3/6) had non-affected parents as compared to 18% (9/55) in the group of children who performed −2 standard deviations below the norm average.

To conclude, children with SLI, no matter if they had affected or non-affected parents, performed significantly poorer on NWR Binary as compared to the controls. However, we additionally found that growing up with one or two parents affected with LLP was significantly associated with poorer results on NWR in children with SLI, irrespective of the number of parents, or the gender of the parent(s) that was/were affected.

### Association between the Performance of the Children with SLI on NWR and Parents’ Level of Education

The level of education for the parents of the children with SLI was divided into three categories as follows: elementary school 14%; completion of upper secondary school 48%; and higher education (i.e., university studies) 38%. There was no difference in the NWR Binary performance when we grouped the children with SLI according to the mothers’ level of education (F(2.58) = .266, p = .768) and the fathers’ level of education (F(2.57) = 1.279, p = .286), as determined by ANOVA. The proportion of parents with a higher level of education was significantly higher in the families where the parents did not have LLP, with 75% of the mothers (Chi^2^ 6.98, p = .008) and 67% of the fathers (Chi^2^ 4.53, p = .033) having taken higher education. This is compared to the group of parents with LLP in which 29% of the mothers and fathers respectively had a higher education. There was a significant association between the prevalence of LLP and level of education for mothers (r = −.289, p = .024) and for fathers (r = −.413, p = .001), on the basis of a Spearman bivariate correlation, two-tailed analysis.

## Discussion

This is the first comprehensive study reporting performance on NWR in Swedish-speaking school-age children. The study is also providing evidence-based support for NWR as a clinical marker for SLI in a Swedish-speaking population aged 8–12 years. Our study suggests a cut-off at −2 standard deviations based on binary scoring of NWR, which correctly identifies 90.2% of the children with SLI and 97.7% of typically developing children (TD). Furthermore, our findings showed that performance on NWR was insensitive to age, gender, non-verbal IQ, and parents’ level of education in the group of children with SLI. Further, having parents with LLP was associated with lower scores on NWR in children with SLI. The difference between the SLI subgroups with affected and unaffected parents was large (d = 0.7). In addition, the magnitude of the effect size in relation to the TD group was substantially larger (d = 2.47) for the SLI subgroup with affected parents as compared to the subgroup with unaffected parents (d = 1.57).

A clinical marker is a measurable deficit characterizing a particular disorder or condition; in other words, it distinguishes between people who have a certain disorder and those who do not. The provision of group difference values from a test does not necessarily equal clinically adequate sensitivity and specificity values for a correct classification [54]. There is, however, no broadly accepted guideline for interpretation of the clinical importance of sensitivity and specificity values [55]. Following the suggested threshold of above 90% for sensitivity and specificity, our results can be considered as clinically “good” [56], given that we found only 2.3% false positives and 9.8% false negatives. Our finding of differences regarding clinical accuracy for different cut-off levels was expected. Our results clearly point to the importance of empirically derived cut-off levels in contrast to commonly used ones such as −1 or −1.5 standard deviations for language measures, for example. Knowledge about a test’s accuracy is clinically crucial, especially in standardized diagnostic assessments. Application of a higher cut-off score in the present study would lead to a significant over-identification of SLI in typically developing children. In clinical assessments, a test score at borderline to a cut-off value must be handled carefully since it risks being less reliable, as, for example, when because of overlap between a target group and controls.

Our finding of 90.2% sensitivity means that almost 10% of the children with SLI scored above the cut-off level. Also, other researchers have reported that not all children with SLI show low performance on NWR [57]–[59]. On the other hand, it is also possible to perform poorly on NWR and yet not develop SLI [60]. In a study of 242 eleven-year-old children from language units in the UK [58], it was found that 61% scored below −1 standard deviation on a test of NWR, and a small number (5.8%, 14/242) of their SLI probands had high scores above 1 standard deviation. The authors speculate that it might be that the high-scoring children had received “more intensive, early or appropriate” phonological intervention. This kind of intervention might have had a positive effect on NWR test scores since the test taps a range of phonological skills typically focused on in speech therapy [61]. Therefore, in the present study, we also investigate sensitivity in the children with SLI based on their speech output status. In the SLI group, when adding up children with normal speech status and children with minor speech output problems (n = 40, 66%), we found 85% sensitivity on the basis of results below −2 standard deviations on NWR Binary. This is still a value of sensitivity that is acceptable [56]. Importantly, more than three quarters of the children with currently completely normal speech status showed extraordinary difficulties (below −2 standard deviations) in NWR as compared to the TD group, which corresponds to a sensitivity of 75%. Normal speech status at the age of 8–12 years does not rule out earlier speech deficits in the child, or that the child had received speech intervention at a younger age. It is well known that speech sound deficits found in preschool-age seldom persist into the later school years [62]. The question is if isolated speech deficits are more often resolved than speech deficits that co-occur with language impairment? Unfortunately, data regarding earlier speech status are lacking in the present study. However, five out of the six children with SLI who have a NWR score above the cut-off point of −2 standard deviations had normal speech status at the time for the participation in our study, and one child had minor speech deficits, (defined as occurrence of substitutions of/r/, or lisping). Interestingly, that child had a history of severe speech deficits until the age of 6–7 years, according to the parents.

In NWR, children’s speech output deficits have been dealt with in different ways depending on the purpose of the study and the targeted age groups. In a study of NWR in Dutch-speaking preschool children [23], phonological errors were treated as correct if the child showed at least 75% correct production of a phoneme on a picture-naming task. These kinds of developmental speech errors are normally not present in Swedish 8–12 year old children [53] and we therefore only accepted correct repetition of the nonword. Another option would have been to exclude children with speech production deficits, an approach that has been applied in previous studies [12], [57], [63]. However, excluding one third of our sample of children with SLI with speech output deficits would have affected the representativeness of our sampling. Compared to a population-based study [64], where the co-occurrence of speech output deficits in children with SLI was found to be 5–8%, we have found a much higher rate of speech output deficits in our sample with SLI. This is probably explained by our sample being a clinical sample of SLI. These samples are known to more commonly include speech sound deficits than do population-based samples [29].

NWR was initially suggested to reflect phonological short-term memory [65], but has been shown to be associated with a range of measures of lower and higher level language processing [15]–[18], [61]. It was, however, not the purpose of the present study to investigate the associations between NWR and other measures. Moreover, poor NWR performance has also been found in children with autism [66], Down’s syndrome [67], and reading impairment [68], [69], though, none of these studies has reported the sensitivity and specificity of NWR. NWR probably captures speech and language deficits that are present in a range of neurodevelopmental disorders, for example in children with autism who also have language impairment [66].

Compared to other studies of clinical accuracy of NWR [7], the present study is based on the largest clinical sample so far. Few previous studies of NWR performance in school-age children report sensitivity and specificity values. One exception is the study by Archibald and Joanisse [70] who, in a population-based study, found low values (70%) for both sensitivity and specificity in school-age children. However, NWR has been shown to be more effective in distinguishing children with SLI from TD children in clinical samples than in epidemiological samples [21]. One possible explanation, is that SLI diagnosed by a speech-language pathologist and SLI classified by an experimental design represent different phenotypes and etiology of SLI (e.g., children with co-occurring SLI and speech sound deficits are more likely to be referred to a speech-language pathologist, than children with SLI only [71]). This might also explain the lower results for sensitivity and specificity in the study by Archibald and Joanisse [70].

The effect size of the difference on NWR performance between the SLI group and the TD group in the present study is similar to several other studies investigating comparable age groups [63], [72], [73]. However, it is hard to compare our results of NWR performance with previous studies. One reason might be that the magnitude of the effect size is related to the version of NWR test being used [7]. Another reason is that we lack additional Swedish data, as there are no previous studies exploring NWR with the same test as in the present study, in a Swedish-speaking sample with SLI. Studies of NWR in Swedish children with SLI have focused on methodological aspects; for example, looking at the construction, scoring, and analysis of a Swedish NWR test [13], [17], [74], [75], as well as on the relationship of NWR with other linguistic and cognitive measures in children with SLI aged four to seven years [76], [77].

In the present study, we have found that children with SLI that grow up with parents with LLP have poorer NWR performance than those who do not have parents with LLP. To our knowledge, no other study has looked at contextual influences on NWR, such as, for example, the aptitudes and attitudes towards language in a family where the parents themselves have language-related problems, and may be struggling with word forms, reading, and writing. The poorer performance on NWR is not necessarily explained by the home language environment. It is, however, highly plausible that linguistic input for children with SLI differs depending on whether parents have LLP or not. We know that parental linguistic input has an impact on SLI children’s language development, but to our knowledge there are no previous studies comparing the home language environment of children with SLI with affected parents to that of children with unaffected parents. However, it is important to remember that a child with SLI will also contribute to, and shape the interaction within a family, because of its own limitations in speech and language abilities [78]. It is well-known that there is a mutual adjustment of communicative behaviors in parent-child interactions where the child has language impairment, so that the interaction pattern is regulated according to the linguistic ability of the child [79]–[81]. It still remains to be explored how this mutual adjustment is affected in families where parents themselves are struggling with language-related problems.

Only when we added up language and/or literacy problems as a family risk component, did we find an association with lower NWR performance in the children with SLI. This did not occur when we analyzed one of the two language-related problems at a time. We believe that parents with language problems and parents with reading problems may contribute to similar home language environments when it comes to language input to the child, such as attitudes to reading. High levels of co-occurrence of language and literacy problems in parents of children with SLI have been reported in previous family history studies [82]. In addition, in the present study LLP in the parents co-occur with low levels of parental education, which is another factor that may have direct and indirect influence on home language environment and children’s development.

The influence of parental language input and its interaction with genetic transmission is complex. We know from twin studies that social environmental influence is larger than the genetic influence on children’s reading ability in families with lower educated parents, than in families with higher educated parents [83]. Furthermore, the genetic influence on language and cognitive development varies with age [84]. This means that heredity, that is, our genetic material, is not determinative, but plays a dynamic role during development together with environmental factors. We may all carry genetic risk variants for disorders that we neither develop nor transmit to our children. As we have shown previously [39], language-related problems were found in about half of the siblings of children with SLI. Therefore, children with unaffected parents may still have siblings as well as grandparents with language-related problems, potentially contributing to genetically and socially inherited language environments that probably differ from what can be found in controls. Reports on NWR as a clinical marker for SLI based on sensitivity and specificity values do not include considerations of genetic or environmental influence on NWR. These kinds of mechanisms cannot be disentangled on the basis of a family aggregation study. Moreover, the limitations of self-reported data need to be acknowledged. For different reasons, people might over- or underreport their language related problems. People might not be aware of their own history of language-related problems or current ones, or they may overestimate the problems they have. In addition, people might not be willing to share this kind of information with an outsider, such as a researcher. One may presume that direct testing of the parents in our study would generate more reliable data, but it was not an option for us to assess the parents for all of the language related diagnoses and problems included in the interview. In a previous family aggregation study [38], the two data collection methods (telephone interviews and direct testing) were compared in first-degree relatives of children with SLI. The authors found that the two different methods revealed similar prevalence rates (35% and 35.5%) of language and literacy problems in the relatives. Importantly, with our choice of method (telephone interviews), we could gather information about 100% of the parents. Still, an investigation of nonword repetition in parents would be of great value in order to analyze association with parents’ own language status and their children’s NWR performance. Interestingly, in a previous study [37] NWR was suggested as a marker of family risk of language impairment, based on NWR being a good discriminator between groups of parents who had children with and without SLI, respectively. As previously mentioned, NWR has been found to be highly heritable and associated with poorer language acquisition [34]. In addition, in a twin study [32], heritability was found to increase with the severity of language impairment. Furthermore, as suggested by Bishop and Hayiou-Thomas [71], there may be different fundamental etiologies for co-occurring SLI and speech sound deficits, as compared to SLI-only. The first is much more heritable, while the second has been shown to be more environmentally driven. Consequently, based on our data on family aggregation, children’s NWR performance and their speech status, we might speculate that the six children with normal NWR in our sample of children with SLI constitute a less genetically influenced phenotype of SLI as compared to the children with poor NWR performance. This will be further investigated in our following studies of genetic risk markers in Swedish SLI.

Another aspect of the family context is the parents’ level of education. Living in a family with parents having a higher level of education has been shown to be indicative of higher expectations for the children’s development, and is also possibly providing a more challenging linguistic and cognitive home environment [45], [85]. The distribution of educational level of the parents to the children with SLI in our study corresponds to the distribution in the general Swedish population (www.scb.se). Not unexpectedly, in the present study, the proportion of parents with a higher level of education was found to be lower in parents’ with LLP, as compared to the unaffected parents. It would be interesting, and possibly add to implications for intervention strategies, if we knew more about the positive mechanisms behind the academic achievements of the 29% parents with LLP who, in spite of their problems, have reached university level. However, even more important than parents’ formal level of education, is the quality of the linguistic, cognitive, social, and emotional stimulation parents are able to provide for their children [86], [87].

### Clinical and Methodological Considerations

In summary, the computer-based NWR test we used in the present study has good potential to distinguish between Swedish-speaking school-age children with SLI and typically developing children. However, since this is the first study investigating NWR as a clinical marker in Swedish SLI, our findings need to be replicated. A limitation to our study is lack of blindness to group membership in the scoring of the NWR test, since language status was known to raters and could have created bias. Another possible limitation may be that non-verbal IQ was assessed with different tests in the participants with SLI and the controls.

Another finding was that, older children with SLI did not perform significantly better on NWR than younger children with SLI in our study (which was the case in the TD group). Lack of developmental change is a core feature of a clinical marker. SLI is considered pervasive and often persistent [88]. Although our study is not longitudinal, the lack of relation between age and NWR performance in the SLI group is interesting. This finding may corroborate earlier findings showing persistently poor NWR performance in school-age children with SLI as in school-age children with resolved language impairment [88]. A lack of improvement of NWR performance in a child may be of great predictive value since the ability to exactly recall how new words sound is crucial for a range of complex language activities during the school years. Furthermore, findings in the present study raise questions about contextual factors that may interact with NWR performance in children with SLI. Parents’ own language-related problems may influence the linguistic input to the child. A clinical ambition of a family-oriented approach requires more knowledge about contextual influences on children’s language processing skills. The counseling provided to families about home training and communicative strategies should be based on a careful survey of the resources in the family.
